# Factors Contributing to the Pre-Elimination of Malaria from Hainan Island, China, 1986–2009

**DOI:** 10.4269/ajtmh.23-0303

**Published:** 2023-10-09

**Authors:** Dingwei Sun, Hongwei Jiang, Guangze Wang, Ximin Hu, Shanqing Wang, Yan Chen

**Affiliations:** ^1^ Hainan Provincial Center for Disease Control and Prevention, Haikou, China;; ^2^ Graduate School of Humanities, Osaka University, Toyonaka City, Japan;; ^3^ Research Institute for Humanity and Nature, Kyoto, Japan

## Abstract

Malaria was endemic in Hainan Island, China, for a lengthy period before its elimination. Here, we aim to gain a better understanding of malaria elimination by assessing and quantifying the relative effects of longitudinal changes in specific antimalarial interventions, socioeconomic development approaches, and malaria vectors on malaria prevalence in Hainan during the 1986–2009 pre-elimination period. Annual data were collected on the incidence of malaria, the number of residents protected by drugs (RPD), the number of residents protected by vector control, the presence of *Anopheles minimus* and *Anopheles dirus*, the annual per capita income of rural residents, major cash crop (rubber plantation) areas, the literacy rate of adult rural residents, and the rate of reinforced concrete house construction in rural areas. Backward stepwise multiple linear regression models were developed to identify the factors associated with the annual malaria incidence (AMI). The AMI declined from 20.3 to 0.8 per 10,000 population from 1986 to 2009; this decrease was significantly associated with an increase in the number of RPD and improved literacy rate among rural adults. The results of this study implied that the sustained distribution of antimalarial drugs and increase in education levels in risk areas significantly impacted the reduction and elimination of malaria infection in Hainan. We suggest that these findings could be applicable to malaria-endemic areas in Southeast Asia with similar natural and socioeconomic environments to Hainan and where malaria incidence has decreased to a low level.

## INTRODUCTION

Malaria is a serious disease that affects tropical regions worldwide.^1^ With substantial achievements in decreasing the malaria burden in many regions, the Lancet Commission on malaria eradication has aspired to eliminate malaria outside of Africa by 2030 and worldwide by 2050.^2^ Malaria has been already eliminated in China, where it was once endemic, by leveraging technology, implementing surveillance strategies, and integrating malaria interventions into the health system.^3^

An estimated 30 million cases of malaria occurred annually in China in the 1940s.^4^ While malaria outbreaks occurred throughout the 1960s and 1970s, steady progress toward curtailing the disease was made, with only 363,000 cases reported in 1986.^5^ An integrated strategy was implemented for malaria control, including antimalarial interventions and socioeconomic developments, and malaria infections continued to drop; in 2009, only 11,119 cases were reported.^6^ Simultaneously, the percentage of the counties with an annual malaria incidence (AMI) below 1 per 10,000 population increased from 83.3 in 1986 to 98.1 in 2009.^5^^,^^7^ In conjunction with the United Nations’ 2000 Millennium Development Goals, in 2010, China’s National Health and Family Commission announced the National Malaria Elimination Action Plan to eliminate malaria by 2020.^8^ The period from 2010 to 2020 is generally referred to as the malaria elimination period. China’s National Malaria Elimination Program developed the “1–3–7” approach to guide malaria elimination activities.^9^ This strategy included case reporting, investigation, and responses outlined with a set of targets identifying responsibilities and actions over a specific time frame. The 1 refers to case reporting within 1 day of diagnosis, the 3 denotes case investigation within 3 days, and the 7 indicates that appropriate measures must be taken to prevent further spread within 7 days.^10^^,^^11^

Hainan Island previously had one of the highest malaria rates in China, with incidence rates exceeding 1,000 per 10,000 in 1955.^12^ Since 1959, various large-scale specific antimalarial interventions have been introduced in Hainan, including indoor residual spraying (IRS), insecticide-treated nets (ITNs), preventive chemotherapy (PC), long-lasting insecticidal nets (LLINs), mass drug administration (MDA), and targeted drug administration (TDA) (Supplemental Materials, pp. 1–2). As a result, the transmission of malaria in Hainan Island has changed dramatically. In 2009, only 685 indigenous cases occurred across the total population of 8.67 million individuals.^13^ Moreover, in 2020, Hainan Province achieved both China’s and the WHO’s goal of malaria elimination: no indigenous malaria cases were reported for 5 consecutive years (2016–2020).

Similar to the situation in other malaria-endemic areas worldwide, socioeconomic developments supported continual intervention and contributed to malaria reduction in Hainan and other areas in China. Previous researchers have summarized three aspects regarding the impact of socioeconomic developments on malaria reduction. First, socioeconomic developments help mitigate the disease by improving living conditions^13^^,^^14^ and the sustainability of effective interventions^15^; reducing mosquito bites, and thus the risk of malaria infection^13^^,^^14^; and providing timely and effective treatment of malaria patients.^16^^,^^17^ Such socioeconomic developments resulted in the natural disappearance of malaria from certain European regions.^18^^,^^19^ Second, socioeconomic developments contributed to the effective scale-up of interventions as sanitary conditions improved, leading to a decline in incidence rates.^20^^,^^21^ Third, socioeconomic developments contributed to the sustainability of antimalarial interventions in some post-elimination countries where the malaria vectors are still present, and even one imported case could trigger a rebound.^19^^,^^20^^,^^22^^,^^23^

Malaria transmission is a dynamic and longitudinal process; therefore, the goal of malaria elimination and eradication cannot be achieved in one step.^2^ Understanding the longitudinal relationship between the incidence of malaria and contributing factors could provide unique and comprehensive insight for the regions still affected by the disease.^19^^,^^20^^,^^24^^–^^26^ Yet, to our knowledge, most previous longitudinal studies focused mainly on the correlation between malaria incidence change and one specific antimalarial intervention or the impacts of a sole socioeconomic development factor on malaria reduction and elimination.^19^^,^^27^^,^^28^ Few systematic analyses of the relative importance of different antimalarial interventions and socioeconomic development factors have been conducted. Moreover, the limited existing longitudinal studies provided insufficient information about the stationarity and co-integration of multiple time-series data, which could raise doubts about the reliability of these longitudinal analyses.^28^^,^^29^

Here, we aimed to better understand the impacts of socioeconomic developments and specific antimalarial interventions on malaria elimination in Hainan. We collected annual data on malaria incidence, antimalarial interventions, and major socioeconomic development factors in Hainan from 1986 to 2009 and analyzed this dataset with time-series analysis methods to understand the longitudinal impacts of socioeconomic developments and specific antimalarial interventions on malaria transmission in the pre-elimination phase. Our results provide insights and knowledge useful for consideration in malaria-endemic regions, especially those in Southeast Asia.

## MATERIALS AND METHODS

### Study area.

**Figure 1.**
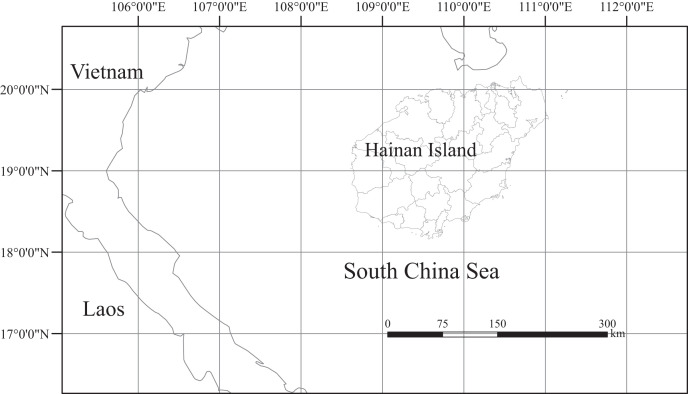
Geographic location of Hainan Island, China. Hainan Island is located between latitudes 18.10°N and 20.07°N and longitudes 108.37°E and 111.03°E.

Hainan Island is located in Southern China (latitude 18.10°–20.07°N, longitude 108.37°–111.03°E, Figure 1) and has a land area of 33,920 km^2^.^30^ The tropical monsoon climate produces an average annual temperature of 22 to 26°C and an average annual rainfall of 1,500 to 2,500 mm.^31^ Hainan Island has distinct wet and dry seasons, with the wet season running from May to October and the dry season running from November to April.^32^ The tropical environment produces an ideal region for vector mosquitoes to breed. Malaria was previously actively transmitted throughout the year in Hainan; historically, the island was once the site of China’s most devastating malaria epidemics.^33^ In 2010, China’s Census reported that the resident population of Hainan Island exceeded 8.67 million.^34^

### Data collection.

Annual data on the malaria incidence change and associated predictive variables were collected for statistical analysis from 1986 to 2009, covering specific antimalarial interventions, vectors, and socioeconomic development factors.^13^^,^^15^^,^^35^^–^^38^ The AMI and intervention factor variables were collected from annual antimalaria reports of each county/city (Hainan Provincial Research Institute for Tropical Disease Control, unpublished data) (Supplemental Materials, pp. 1–2).^38^ The intervention factor variables were residents protected by drugs (RPD) and residents protected by vector control (RPV). RPD represented the annual total number of residents protected by MDA, TDA, and PC. RPV represented the annual total number of residents protected by IRS, ITNs, and LLINs. The vector factor variables were the annual presence of *Anopheles minimus* (PAM) and *Anopheles dirus* (PAD) (Supplemental Materials, pp. 1–2). The socioeconomic development factors were annual per capita income of rural residents (APCI), rubber plantation areas (RPA), the literacy rate of adult rural residents (LRR), and the rate of reinforced concrete house construction in rural areas (RRC); the data were obtained from the Hainan Statistical Yearbook (Supplemental Materials, pp. 1–2).^39^^–^^49^

### Statistical analysis.

**Figure 2.**
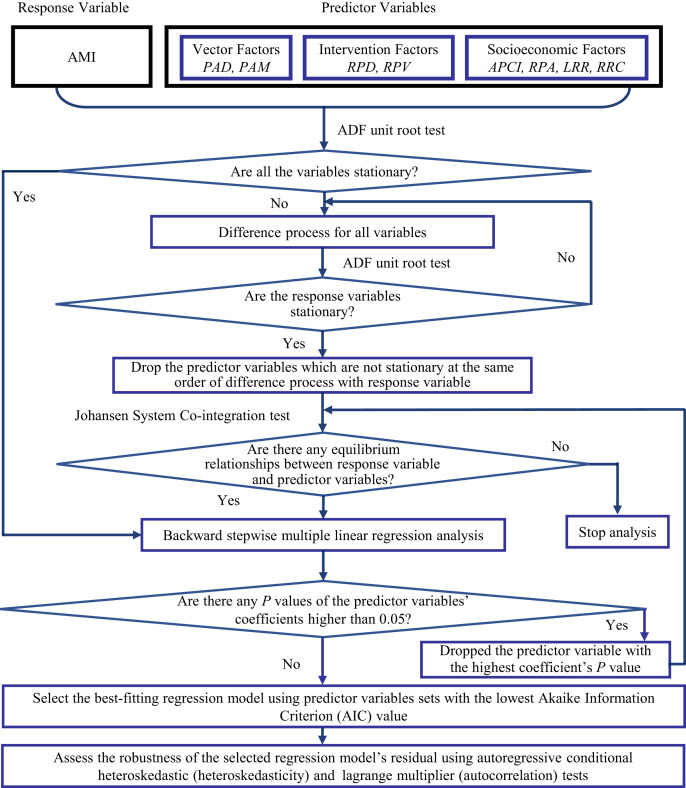
Flow chart of statistical analysis. The response variable was the annual malaria incidence (AMI) and the predictor variables included the presence of *Anopheles minimus* (PAM) and *Anopheles dirus* (PAD) as vector factors; the number of residents protected by drugs (RPD) and the number of residents protected by vector control (RPV) as intervention factors; and the annual per capita income of rural residents (APCI), rubber plantation areas (RPA), literacy rate of adult rural residents (LRR), and rate of reinforced concrete house construction in rural areas (RRC) as socioeconomic factors.

The statistical analysis process is shown in Figure 2. Because all variables were time-series data, their stationarity was tested before the multiple regression analysis was conducted. If the response variable demonstrated stationarity at level or stationarity after first or higher-order differencing, the cointegration between the response variable and candidate predictor variables, which demonstrated stationarity in same-order differencing as response variable, was also tested. Stationarity tests help to determine whether there is a random or definite time trend in the response or predictor variables. The cointegration test helps to determine whether there is a definite relationship between the response variable and predictor variables in the designated period. In the present study, stationarity and cointegration tests were conducted using the augmented Dickey–Fuller unit root test and the Johansen system cointegration test, respectively.

A backward stepwise multiple linear regression analysis was conducted. In this analysis process, the predictor variables with coefficient *P* values greater than 0.05 were dropped stepwise from the regression model by descending *P* values until the *P* values of all predictor variable coefficients were less than 0.05. Each time a predictor variable was dropped, the cointegration between the response variable and the remaining predictor variables was tested. The best-fitting regression model was selected using the predictor variable sets with the lowest Akaike information criterion (AIC) value.

Heteroskedasticity and autocorrelation were tested using autoregressive conditional heteroskedastic (ARCH) and lagrange multiplier (LM) tests, respectively, to assess the robustness of the selected regression model’s residual. The time lag of these two tests was set at 1 to 4 years, and the significance level was 0.05. We estimated whether heteroskedasticity and autocorrelation existed in the residual of the selected regression model based on the significant *P* value in the *F* statistics and observations × *R*^2^ of the ARCH or LM model with a time lag from 1 to 4 years.

We used the cross-correlation function to calculate the cross-correlation coefficients between the response variable and all predictor variables with a time lag from 0 to 4 years to determine whether a time lag exists between predictor variables and the response variable in the selected model. If the response variable was not stationary, we calculated the cross-correlation coefficients using the difference-processed variables, which demonstrated stationarity. The significance level was set to 0.05, and the cross-correlation coefficient value was 0.40.

All statistical analyses were conducted using Eviews version 12 (HIS Global, Inc.)

## RESULTS

### Changes in the antimalarial intervention program and socioeconomic developments in Hainan Island.

**Figure 3.**
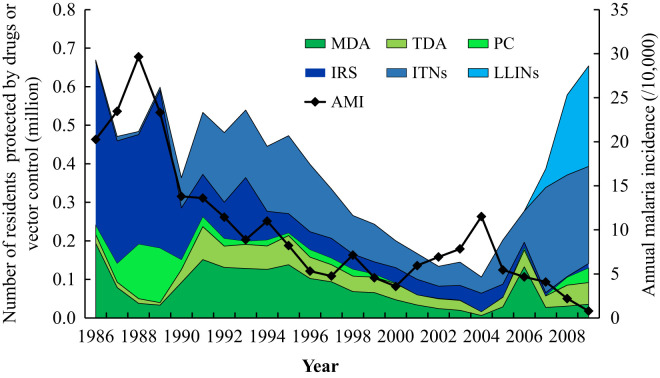
Annual malaria incidence (AMI), the annual number of residents protected by drugs (RPD), and the annual number of residents protected by vector control (RPV) on Hainan Island, 1986–2009. The left Y-axis represents the RPD or RPV, and the right Y-axis represents the AMI. The RPD value includes protection through mass drug administration (MDA), targeted drug administration (TDA), and preventive chemotherapy (PC). The RPV value includes those protected by indoor residual spraying (IRS), insecticide treated nets (ITNs), and long-lasting insecticide nets (LLINs).

**Figure 4.**
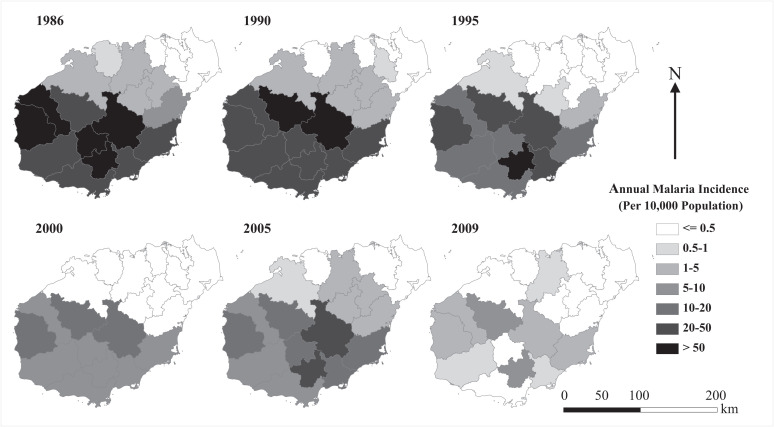
Geographic distribution of annual malaria incidence (per 10,000 population) on Hainan Island, 1986–2009. The counties are color-coded by the annual malaria incidence.

The trend of the AMI change and the number of residents protected by antimalarial intervention programs from 1986 to 2009 is shown in Figure 3. In general, the AMI decreased from a peak of more than 20 per 10,000 population in 1988 to less than 1 per 10,000 population in 2009, although a rebound was observed in the early 2000s. However, the number of residents protected by antimalarial intervention programs fluctuated. From 1986 to 1996 and 2007 to 2009, the yearly total numbers of RPD and RPV were ∼0.4 million or higher, whereas total RPD and RPV numbers decreased from 0.33 million in 1997 to 0.11 million in 2004. Similarly, as shown in Figure 4, the number of counties with an AMI exceeding five per 10,000 population decreased from 11 in 1986 to two in 2009, corresponding to an overall decrease in RPD and RPV. An endemic malaria rebound was also observed in 2000 and 2005.

**Figure 5.**
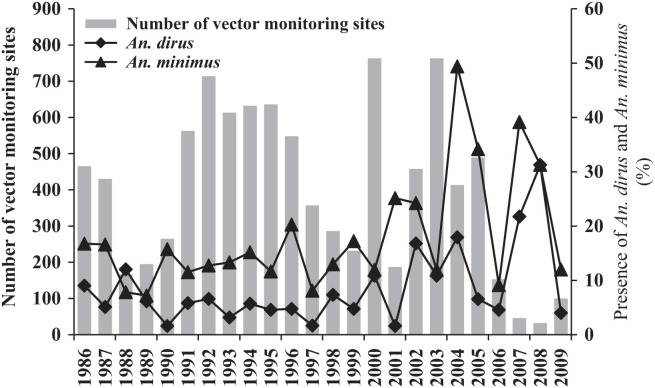
Yearly change in the presence (percentage) of *Anopheles dirus* and *Anopheles minimus* at the vector monitoring sites on Hainan Island, 1986–2009. The left Y-axis represents the number of vector monitoring sites and the right Y-axis represents the percentage of vectors present.

The monitoring of major vectors from 1986 to 2009 is shown in Figure 5. The total number of vector monitoring sites in this period was 8,932, with a mean of 372 ± 202 sites per year. *An. dirus* and *An. minimus* were observed at an average of 8.5% ± 7.2% and 18.3% ± 10.7% of the sites, respectively. In general, *An. minimus* was more common than *An. dirus* at the vector monitoring sites in Hainan Island. No clear vector change trend was observed from 1986 to 2009.

**Figure 6.**
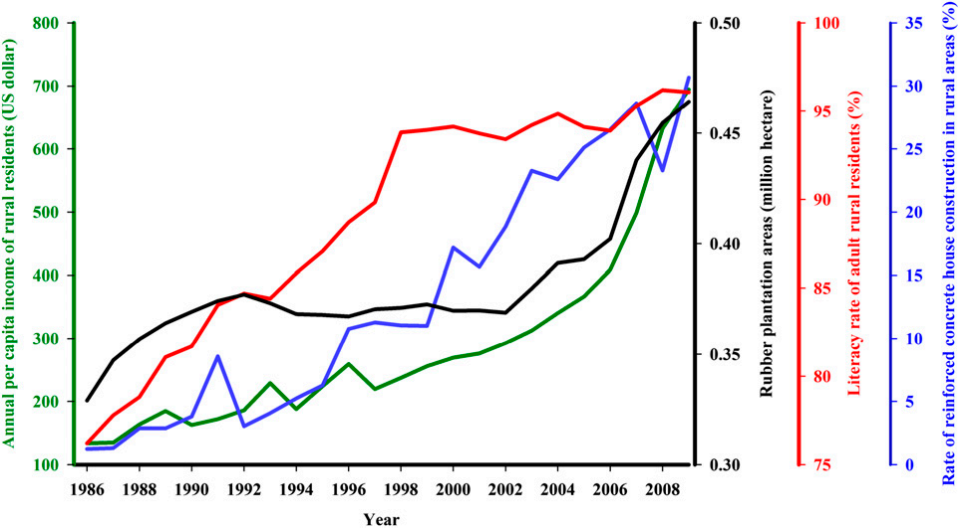
Socioeconomic developments on Hainan Island, 1986–2009. The left Y-axis represents the annual per capita income of rural residents (APCI, green line). The three right Y-axes, from left to right, represent the rubber plantation areas (RPA, black line), rubber being the major cash crop in the malaria-endemic area of Hainan Island; the literacy rate of adult rural residents (LRR, red line); and the rate of reinforced concrete house construction in rural areas (RRC, blue line), respectively.

As shown in Figure 6, the socioeconomic factors have improved greatly from 1986 to 2009. The APCI increased from 134 to 694 dollars, the RRC increased from 1% to 31%, and LRR increased from 76% to 96%. The RPA increased from 0.33 to 0.46 million hectares.

### Statistical analysis results.

The stationarity of the response variable AMI and all candidate predictor variables were tested using the augmented Dick–Fuller unit root test. All variables were not stationary (*P* > 0.05) at level. The response variable and candidate predictor variables except for RPA demonstrated stationarity (*P* < 0.05) at first-order of differencing (Table 1). As a result, the candidate predictor variable RPA was excluded from backward stepwise multiple linear regression analyses.

**Table 1:** Stationarity test with augmented Dickey–Fuller unit root test results

Variable/difference-processed variable	*t* statistic	*P* value
AMI at level	−1.70	0.084
First difference of AMI	−4.14	< 0.001***
PAD at level	−1.80	0.667
First difference of PAD	−6.65	< 0.001***
PAM at level	−2.07	0.529
First difference of PAM	−10.32	< 0.001***
RPD at level	−2.79	0.214
First difference of RPD	−7.64	< 0.001***
RPV at level	−0.47	0.977
First difference of RPV	−5.97	< 0.001***
RRC at level	−3.37	0.081
First difference of RRC	−7.40	< 0.001***
APCI at level	3.03	1.000
First difference of APCI	−3.78	0.038*
LRR at level	−2.37	0.159
First difference of LRR	−4.57	0.008**
RPA at level	0.52	0.998
First difference of RPA	−1.02	0.918

Using the augmented Dickey–Fuller unit root test, the stationarity of the variables or the difference-processed variables (if the response variable was nonstationary) was tested, including annual malaria incidence (AMI), number of residents protected by drugs (RPD), number of residents protected by vector control (RPV), annual per capital income of rural residents (APCI), rubber plantation areas (RPA), literacy rate of adult rural residents (LRR), rate of reinforced concrete house construction in rural areas (RRC), presence of *Anopheles dirus* (PAD), and presence of *Anopheles minimus* (PAM). *P < 0.05. **P < 0.01. ***P < 0.001.

The backward stepwise multiple linear regression analyses between response variable AMI and predictor variables RPD, RPV, APCI, LRR, RRC, PAD, and PAM were conducted based on the stationarity test results. In each step, the predictor variable with a coefficient *P* value > 0.05 was dropped from the analysis in descending order (Supplemental Materials, pp. 3, Supplemental Table 1). The cointegration test revealed at least one cointegration between the response variable and predictor variables in each step (Supplemental Materials, pp. 4, Supplemental Table 2). The backward stepwise multiple linear regression analyses revealed the estimated coefficients of RPD and LRR to be −36.97 (*t* = −2.54, *P* = 0.019) and −1.35 (*t* = −9.09, *P* < 0.001), respectively; both predictor variables positively significantly contributed to the reduction in the AMI from 1986 to 2009 (log likelihood = −59.61; *F* statistic = 36.06, *P* < 0.001; AIC = 5.30) in the best-fitting model (Table 2).

**Table 2:** Multiple linear regression analysis results

Predictor variables	Estimated coefficient	Standard error	*t* statistic	*P* value
RPD	−36.97	14.54	−2.54	0.019*
PAD	0.15	0.10	1.42	0.170
LRR	−1.35	0.15	−9.09	< 0.001***
C	134.54	14. 68	9.13	< 0.001***

The best-fitting model was estimated using backward stepwise multiple regression analysis. The number of residents protected by drugs (RPD), presence of *Anopheles dirus* (PAD), and literacy rate of adult rural residents (LRR) were included in this regression model (log likelihood = −59.61, *F*-statistic = 36.06, *P* value < 0.001, Akaike information criterion (AIC) = 5.30). *P < 0.05. **P < 0.01. ***P < 0.001.

The residual heteroskedasticity (time lag range: 1–4 years) of the selected regression model in the backward stepwise multiple linear regression analyses was assessed using the ARCH test. The AIC values of the ARCH model with a time lag ranging from 1 to 4 years were 7.92 (*F* = 0.29, *P* = 0.597; observations × *R*^2^ = 0.31, *P* = 0.577), 8.01 (*F* = 0.51, *P* = 0.607; observations × *R*^2^ = 1.13, *P* = 0.569), 6.69 (*F* = 2.21, *P* = 0.125; observations × *R*^2^ = 5.88, *P* = 0.117), and 6.66 (*F* = 1.10, *P* = 0.392; observations × *R*^2^ = 4.54, *P* = 0.338) (Table 3). All the *P* values of the *F* statistics and observations × *R*^2^ of ARCH models were higher than 0.05. The results indicated no heteroskedasticity in the residual of the selected regression model.

**Table 3:** Autoregressive conditional heteroskedasticity test results

Model (lag length years)	Akaike information criterion	*F* statistic (*P* value)	Observation × *R*^2^ (*P* value)
ARCH (4)	6.66	1.10 (0.392)	4.54 (0.338)
ARCH (3)	6.69	2.21 (0.125)	5.88 (0.117)
ARCH (2)	8.01	0.51 (0.607)	1.13 (0.569)
ARCH (1)	7.92	0.29 (0.597)	0.31 (0.577)

ARCH = Autoregressive conditional heteroskedasticity.

The ARCH test was used to test the heteroskedasticity of the residual in the selected regression model. The time lag of the ARCH test was set to 1, 2, 3, and 4 years. The significance levels of the *F* statistic, and observation × *R*^2^ values were set to 0.05.

The residual’s autocorrelation (time lag range: 1–4 years) in the selected regression model in the backward stepwise multiple linear regression analyses was assessed using the LM test. The AIC values of the LM models with a time lag ranging from 1 to 4 years were 5.38 (*F* = 0.006, *P* = 0.941; observations × *R*^2^ = 0.007, *P* = 0.933), 5.28 (*F* = 1.82, *P* = 0.191; observations × *R*^2^ = 4.03, *P* = 0.133), 5.31 (*F* = 1.52, *P* = 0.246; observations × *R*^2^ = 5.07, *P* = 0.167), and 5.29 (*F* = 1.67, *P* = 0.206; observations × *R*^2^ = 7.07, *P* = 0.133) (Table 4). All the *P* values of the *F* statistics and observations × *R*^2^ of LM models were higher than 0.05. Thus, no autocorrelation was present in the residual of the selected regression model.

**Table 4:** Lagrange multiplier test results

Model (lag length, years)	Akaike information criterion (AIC)	F-statistic (*P* value)	Observation × R-squared (*P* value)
LM (4)	5.29	1.67 (0.206)	7.07 (0.133)
LM (3)	5.31	1.52 (0.246)	5.07 (0.167)
LM (2)	5.28	1.82 (0.191)	4.03 (0.133)
LM (1)	5.38	0.006 (0.941)	0.007 (0.933)

LM = Lagrange multiplier

The LM test was used to test the autocorrelation of the residual in the selected regression model. The time lag of the LM test was set to 1, 2, 3, and 4 years. The significance levels of the *F* statistic, and observation × *R*^2^ values were set to 0.05.

The cross-correlation analysis revealed no significant cross-correlation coefficients between the response variable and predictor variables in the best-fitting regression model at a time lag ranging from 0–4 years (Supplemental Materials, pp. 5, Supplemental Table 3).

## DISCUSSION

The stationarity test showed the indispensability of time-series statistical methods in longitudinal malaria research. As previously mentioned, if the original or difference-processed time-series data used in statistical analysis are not stationary, we cannot determine whether significant correlations between variables are spurious or genuine. In the present study, we excluded RPA from the candidate predictor variables based on the stationarity test results. However, to our knowledge, most previous longitudinal malaria studies assessing time-series data either failed to consider these issues sufficiently or did not adequately explain the data analysis process.^27^^–^^29^ Similarly, in some previous longitudinal malaria studies^28^^,^^29^ using multiple regression analyses, no information was provided regarding the cointegration of the response variable and predictor variables. If so, the authors of these studies cannot statistically reject that their results were not spurious regression. Thus, we strongly recommend applying appropriate multiple time-series statistical analysis methods in longitudinal malaria studies, thereby reducing spurious conclusions.

Furthermore, the present study is one of few longitudinal studies to consider systematically the impacts of antimalarial intervention and socioeconomic factors on the AMI with multiple regression analysis. Although some previous studies have been conducted on the correlation between malaria incidence and intervention factors or socioeconomic factors separately and indicated that socioeconomic development contributed to the reduced incidence, the major contributing factors were not clarified.^19^^,^^27^^,^^28^ One longitudinal study in Yunnan, China, used a structural time-series model to analyze the effects of several malaria intervention methods on reducing malaria incidence.^50^ Unfortunately, the effects of socioeconomic factors were not assessed. Consequently, we recommend using systematic analysis methods (e.g., regression model) to clarify the major factors contributing to longitudinal malaria incidence reduction, improving the efficiency of limited resources distribution, and stressing the most effective control strategies.

Our multiple regression analysis showed that the number of RPD was negatively significantly associated with the change in the AMI. However, a similar association was not observed with the number of RPV. In other words, RPD played a key role in AMI reduction from 1986 to 2009. However, the number of RPV in the same period (except for in 2006) accounted for more than 50% of the number of residents protected by the antimalarial intervention program (Figure 3). This finding is likely due to the source of malaria infection during this period. On the basis of infection reports submitted by 10 malaria monitoring stations around Hainan Island from 2000 to 2009, 2,882 of 3,182 (90.6%) new cases were infected with malaria in remote mountain zones.^38^ Because most infections occurred outside of villages, the contribution of vector control factors in the residence area was limited during this period. Similar effects of MDA, which accounted for 53.8% of the total number of RPD from 1986 to 2009 in Hainan Island, on the reduction of the AMI were reported in very low to low-endemicity areas in Southeast Asian countries.^51^^–^^53^ Although these studies revealed reduced *Plasmodium* prevalence in the target population after MDA, the effects were not sustained. For example, in a cluster randomized trial evaluating MDA for *P*. *falciparum* malaria in several Southeast Asian countries, after 9 months of MDA, the *P. falciparum* prevalence increased from 0.4% to 3.3% in MDA villages and from 5.1% to 6.1% in control villages.^53^ Similarly, from 2000 to 2003, when the number of RPD, including MDA and TDA, was alleviated greatly, the AMI rebounded substantially in Hainan Island (Figure 3). Overall, our results imply that the sustained number of RPD played a major role in the continual AMI reduction in Hainan Island from 1986 to 2009, during which the AMI decreased to a very low level.

The regression analysis showed that the number of LRR was significantly negatively associated with AMI. However, the other socioeconomic factors, such as APCI and RRC, did not significantly associate with the AMI in the same way as the number of LRR. These results probably arose from the provision of free malaria testing and the free drug policy. This policy offset some of the effects of the increase in APCI. However, as mentioned earlier, the main infection source was no longer a residence zone but a remote mountain zone. As a result, the effects of RRC improvement, which can protect residents from vector mosquito biting, have also been offset. Different from APCI and RRC, LRR was associated with the target residents’ capacity to learn and understand antimalaria-related information along with their attitudes and practices. According to the reports from the studies in Cameroon, education level is positively associated with knowledge about malaria transmission and its causative agent and the use of preventive methods.^54^^,^^55^ Additionally, associations between education level and malaria treatment-seeking behavior have also been reported. For example, Birhanu and colleagues reported that the time interval between the onset of fever and first care-seeking of not-attended-school informants was significantly longer than that of attended-school informants in the endemicity area of southwest Ethiopia.^56^ Additionally, Swain and colleagues reported that patients or guardians with higher education levels had significantly better drug adherence compared with the no education group in the northeast states of India.^57^ The increase in LRR from 1986 to 2009 improved the residents’ capacity to accept antimalaria knowledge education. In fact, in 1994, the provincial department of public health initiated a malaria control trial to strengthen health education in the endemicity area in southeast Hainan Island. During the 3-year trial, the rate of bed-net use in the targeted population increased from 26.8% to 72.6%, and the annual incidence of malaria parasites declined from 3.5% in 1994 to 0.8% in 1997.^58^ Most of the malaria endemicity areas at the time were in the least developed areas, such as Hainan Island, many residents had never attended school or had a relatively low education level; thus, our results highlight the value of eliminating illiteracy and strengthening health education as important tools to prevent and control malaria infection.

The regression analysis also indicated that the PAM and the PAD did not significantly associate with the AMI from 1986 to 2009. This finding differed from our assumption that PAM or/and PAD would positively associate with AMI changes. Moreover, no clear decrease in the PAD or PAM was observed (Figure 5). Two possible reasons could explain this result. First, the main infection source has changed to remote mountain zones, and the PAM and PAD collected around the residence zone naturally did not come into contact with the malaria infection source. Second, similar to the reports from northern Cambodia by Vantaux and colleagues, landscape changes after economy-driven human activity are likely to result in large changes in *Anopheles* communities over time and space and in the frequency of opportunistic feeding (anthropophilic or zoophilic) or day-biting behavior, highlighting the key mechanism driving residual human malaria transmission in Cambodia.^59^ From 1986 to 2009, the socioeconomic factors of rural areas in Hainan Island, such as APCI and RPA, have increased greatly (Figure 6); in particular, RPA, which is the main cash crop in malaria-endemic areas, has impacted the landscape greatly. However, the association between land use change and the spatial distribution change of malaria vectors has not been elucidated. Future research on malaria elimination should focus on the temporal-spatial change in vector distribution.

Our study has some limitations. Because of historical data availability, the targeted period, in which the malaria incidence decreased to low or very low levels (Figure 3), was relatively short. Moreover, we only included annual records in our statistical analysis because of data availability. This data deficiency likely affected the precision of the analysis because in some cases, the AMI and some predictive factors changed monthly or across several months. This likely explains why significant cross-correlations were not observed between the AMI and predictor variables in the selected regression model.

## CONCLUSIONS

This was one of few longitudinal studies on the major factors contributing to malaria elimination. Our results highlighted the importance of time-series analysis methods in longitudinal studies. Integrating the antimalarial invention and socioeconomic factors into a time-series regression model allows for advances in assessing the relative importance of both factors in the relatively long period before malaria elimination. Furthermore, our study clarified that the sustained distribution of antimalarial drugs (RPD) and an increased education level (LRR) played important roles in malaria elimination. Nevertheless, the impacts of vector control on malaria transmission cannot be neglected. The present results may represent the synergistic effects of chemoprevention and vector control. We therefore suggest that the results found here could be applicable to malaria-endemic areas in Southeast Asia, where the malaria incidence has decreased to a low level and the natural and socioeconomic environments are similar to those of Hainan Island.

## Supplemental Materials


Supplemental materials
