# Supplementary material for: Factors Contributing to the Pre-Elimination of Malaria from Hainan Island, China, 1986–2009
Source: Am J Trop Med Hyg. 2023 Oct 9;109(5):1063–71. doi: 10.4269/ajtmh.23-0303 (PMC10622475; doi:10.4269/ajtmh.23-0303)
Supplement: Supplementary file 1 [file tpmd230303.SD1.pdf]

Title: Supplementary material covering specific malaria interventions and socioeconomic developments from 1986–2009 in Hainan Island

1. Specific malaria intervention

High instances of malaria occur during the wet season (May to October) in Hainan Island, which is why seasonal antimalaria programs were implemented in endemic areas of the island during this period. Seasonal antimalaria programs included vector monitoring and a series of interventions, including Mass Drug Administration (MDA), Targeted Drug Administration (TDA), Preventive Chemotherapy (PC), Indoor Residual Spraying (IRS), Insecticide Treated Nets (ITNs), and Long-Lasting Insecticide Nets (LLINs). In the statistical analysis, the variables represented the total population covered by the corresponding interventions.

MDA: Target villages in Hainan Island for MDA included villages with an incidence rate of 5% or higher, reports of main vectors, or an abrupt increase in the malaria incidence rate. Each resident included in the MDA was required to undergo a full treatment course of antimalarial medicine during a designated period. However, pregnant women, infants (younger than 12 months), and other residents with medicine contraindication reasons were excluded. Regarding the 1986–1989 MDA data, because the authors could not find the number of residents who took the full course of antimalarial treatment and the local control team implemented the same work protocols as in 1990, the authors estimated the total number undergoing MDA in each corresponding year using the proportion who underwent a full course of antimalarial treatment in 1990 and the total number of residents who accepted antimalaria treatment (including those who did not finish the full course).

TDA: Targets of TDA included residents infected with the *P. falciparum* parasite within 1 year or the *P. vivax* parasite within the past 2 years. TDA also included residents whose blood tests were positive and who were living next door to positive individuals. Each resident included in the TDA was required to undergo a full treatment course of antimalarial medicine in a designated period. The total number targeted with TDA from 1986–1989 was estimated using the method described for MDA.

PC: Targets of PC were residents who lived in the mountain zone around their village during the wet season. They were required to undergo a full treatment course of antimalarial medicine in a designated period, regardless of whether they were infected with malaria.

IRS, ITNs, and LLINs: These variables are the main vector control interventions in Hainan Island. From 1986 to the early 1990s, IRS was the main vector control intervention. Pyrethroids insecticides approved by the WHO, such as deltamethrin, were widely used. From the 1990s to 2007, IRS and ITNs were both used. IRS was used in villages where bednet usage was lower than 70%; otherwise, ITNs were used. ITNs used two kinds of insecticides: deltamethrin and lambda-cyhalothrin. Since 2007, LLINs have been introduced with the support of the Global Fund to Fight AIDS, Tuberculosis, and Malaria (GFATM).

RPD and RPV: Residents protected by drugs (RPD) includes all residents taking MDA, TDA, and PC. Residents protected by vector control (RPV) covers the total population using IRS, ITNs and LLINs.

PAD, PAM, and Vector monitoring: In each wet season approximately 400 villages (372 ± 202, n = 24) were selected as vector monitoring sites. The presence of *Anopheles dirus* (PAD) and *Anopheles*

minimus (PAM) was calculated by the following equations. Presence (%) = Number of vector monitoring sites with reports of *An. dirus* or *An. minimus* / total number of vector monitoring sites

2. Socioeconomic developments

Using the data published in the Hainan Provincial Statistical Yearbook, we collected and/or calculated the following socioeconomic development variables:

APCI: (Annual per capita Income of rural residents): using the annual average exchange rate of Renminbi published by the National Bureau of Statistics of China, we converted the annual per capita income of rural residents published in the Hainan Provincial Statistical Yearbook into US dollars.

LRR (Literacy rate of adult Rural Residents): We estimated the LRR using the total number of illiterate adult members (age of 15 and over) reported by yearly random-sampling rural family surveys in Hainan. Due to the change in the statistical criterion of the Chinese government, the number of illiterate adult rural residents from 2000 onward excluded those aged 15.

The literacy standard of the Chinese central government states that a rural resident should be able to read 1500 Chinese characters, and an urban resident 2000; both rural and urban residents should be able to read and understand simple and popular newspapers, keep simple accounts, and write simple business letters. [1] Knowledge about poverty alleviation, agriculture technology, health education, and family planning is highlighted in the government guidelines for assessing literacy. [2]

RRC (Rate of reinforced concrete house construction in rural areas): Using data from the Hainan Provincial Statistical Yearbook, we calculated the proportion of per capita housing space within reinforced concrete structures in the rural area of Hainan Island.

RPA (Rubber Plantation Area): These data were collected directly from the Hainan Provincial Statistical Yearbook.

Reference

1. Chinese Central Government, 1988. Regulation on the fight against illiteracy. Available at: https://www.gov.cn/zhengce/2020-12/25/content_5573970.htm. Accessed 11 Jul 2023. (in Chinese)
2. Chinese Central Government, 2007. Guideline from the Ministry of Education and 12 other departments on further strengthening literacy efforts. Available at: http://www.moe.gov.cn/s78/A06/jcys_left/moe_706/201001/t20100129_89050.html. Accessed 11 Jul 2023. (in Chinese)

Table 1

Backward stepwise multiple linear regression analysis results

Model	Response variable; Predictor variables	AIC	R-squared	Log likelihood
1	AMI; APCI, RRC, LRR, PAD, PAM, RPD, and RPV	5.51	0.86	−58.14
2	AMI; RRC, LRR, PAD, PAM, RPD, and RPV	5.43	0.86	−58.15
3	AMI; LRR, PAD, PAM, RPD, and RPV	5.37	0.86	−58.42
4	AMI; LRR, PAD, PAM, and RPD	5.32	0.85	−58.88
5	AMI; LRR, PAD, and RPD	5.30	0.84	−59.61
6	AMI; LRR and RPD	5.31	0.83	−60.77

Using backward stepwise multiple linear regression analyses between the response variable Annual Malaria Incidence (AMI) and predictor variables, including residents protected by drugs (RPD), residents protected by vector control (RPV), annual per capita income of rural residents (APCI), literacy rate of adult rural residents (LRR), rate of reinforced concrete house construction in rural areas (RRC), presence of *Anopheles dirus* (PAD), and presence of *Anopheles minimus* (PAM) were conducted. The predictor variable with the highest coefficient *P* value was dropped from the analysis in descending order. Model 5, of which Akaike information criterion (AIC) value was the lowest, was selected as the best-fitting model.

Table 2
Johansen system co-integration test results

Response variable and predictor variables in each step of the multiple regression analysis	Hypothesized number of co-integration equations	Trace statistic	Critical value (α = 0.05)	*P* value
AMI, APCI, RRC, LRR, PAD, PAM, RPD, RPV	None *	241.37	159.53	< 0.001
	At most 1 *	152.29	125.62	< 0.001
	At most 2	96.23	95.75	0.129
	At most 3	60.20	69.82	0.418
	At most 4	34.88	47.86	0.705
	At most 5	20.45	29.80	0.661
	At most 6	8.05	15.50	0.648
	At most 7	2.83	3.84	0.096
AMI, RRC, LRR, PAD, PAM, RPD, RPV	None *	141.30	125.62	0.004
	At most 1	89.03	95.75	0.133
	At most 2	53.38	69.82	0.488
	At most 3	28.76	47.86	0.780
	At most 4	14.37	29.80	0.819
	At most 5	3.72	15.50	0.925
	At most 6	0.47	3.84	0.493
AMI, LRR, PAD, PAM, RPD, RPV	None *	119.09	95.75	0.001
	At most 1*	70.19	69.82	0.047
	At most 2	41.36	47.86	0.178
	At most 3	18.25	29.80	0.548
	At most 4	7.38	15.50	0.534
	At most 5	1.16	3.84	0.282
AMI, LRR, PAD, PAM, RPD	None *	79.60	69.82	0.007
	At most 1*	52.77	47.86	0.016
	At most 2	27.97	29.80	0.080
	At most 3	11.61	15.50	0.177
	At most 4	2.38	3.84	0.123
AMI, LRR, PAD, RPD	None *	55.03	47.86	0.009
	At most 1	28.79	29.80	0.065
	At most 2	12.39	15.50	0.139
	At most 3	2.39	3.84	0.122
AMI, LRR, RPD	None *	38.03	29.80	0.004
	At most 1	12.16	15.50	0.150
	At most 2	2.42	3.84	0.120

Using the Johansen system co-integration test, the co-integration of the response variable annual malaria incidence (AMI) and predictor variables including residents protected by drugs (RPD), residents protected by vector control (RPV), annual per capita income of rural residents (APCI), literacy rate of adult rural residents (LRR), rate of reinforced concrete house construction in rural areas (RRC), presence of *Anopheles dirus* (PAD), and presence of *Anopheles minimus* (PAM) for each step of the multiple regression analysis was tested. * Denotes rejection of the hypothesis at the 0.05 level.

Table 3

The cross-correlation coefficients between the response variable and the predictor variables in the best-fitting regression model

Time Lag (Years)	Predictor Variables		
	RPD	LRR	PAD
0	−0.06	0.17	0.35
1	−0.10	−0.31	−0.12
2	−0.23	−0.18	0.02
3	0.28	0.12	−0.001
4	−0.05	0.06	0.01

The cross-correlation coefficients between the response variable annual malaria incidence (AMI) and the remaining predictor variables, i.e., literacy rate of adult rural residents (LRR), residents protected by drug (RPD), and presence of *Anopheles dirus* (PAD), were calculated with a time lag from 0 to 4 years. No significant cross-correlation was found.
